# Tobacco Smoke and Risk of Childhood Acute Non-Lymphocytic Leukemia: Findings from the SETIL Study

**DOI:** 10.1371/journal.pone.0111028

**Published:** 2014-11-17

**Authors:** Stefano Mattioli, Andrea Farioli, Patrizia Legittimo, Lucia Miligi, Alessandra Benvenuti, Alessandra Ranucci, Alberto Salvan, Roberto Rondelli, Corrado Magnani, on behalf of the SETIL Study Group

**Affiliations:** 1 Department of Medical and Surgical Sciences (DIMEC), University of Bologna, Bologna, Italy; 2 Unit of Occupational Medicine, S.Orsola-Malpighi University Hospital, Bologna, Italy; 3 Occupational and Environmental Epidemiology Unit, ISPO Cancer Prevention and Research Institute, Florence, Italy; 4 Occupational and Environmental Epidemiology Unit, ISPO Cancer Prevention and Research Institute, Florence, Italy; 5 Cancer Epidemiology Unit - Department of Translational Medicine, CPO Piemonte and University of Eastern Piedmont, Novara, Italy; 6 Currently retired, IASI-CNR, Rome, Italy; 7 Paediatric Oncology-Haematology “Lalla Seràgnoli”, Policlinico S.Orsola-Malpighi, Bologna, Italy; Gentofte University Hospital, Denmark

## Abstract

**Background:**

Parental smoking and exposure of the mother or the child to environmental tobacco smoke (ETS) as risk factors for Acute non-Lymphocytic Leukemia (AnLL) were investigated.

**Methods:**

Incident cases of childhood AnLL were enrolled in 14 Italian Regions during 1998–2001. We estimated odds ratios (OR) and 95% confidence intervals (95%CI) conducting logistic regression models including 82 cases of AnLL and 1,044 controls. Inverse probability weighting was applied adjusting for: age; sex; provenience; birth order; birth weight; breastfeeding; parental educational level age, birth year, and occupational exposure to benzene.

**Results:**

Paternal smoke in the conception period was associated with AnLL (OR for ≥11 cigarettes/day  = 1.79, 95% CI 1.01–3.15; P trend 0.05). An apparent effect modification by maternal age was identified: only children of mothers aged below 30 presented increased risks. We found weak statistical evidence of an association of AnLL with maternal exposure to ETS (OR for exposure>3 hours/day  = 1.85, 95%CI 0.97–3.52; P trend 0.07). No association was observed between AnLL and either maternal smoking during pregnancy or child exposure to ETS.

**Conclusions:**

This study is consistent with the hypothesis that paternal smoke is associated with AnLL. We observed statistical evidence of an association between maternal exposure to ETS and AnLL, but believe bias might have inflated our estimates.

## Introduction

Acute leukemia is the most common childhood cancer; acute lymphoblastic leukemia (ALL) accounts for 75–80% of total cases of childhood leukemia, acute non-lymphocytic leukemia (AnLL) for about 20%. [1] Established risk factors, such as exposure to ionizing radiations and genetic syndromes, explain no more than 10% of cases; [2] Suggested risk factors include: car exhaust fumes, pesticides, non-ionizing radiation, pets, antiepileptic drugs, maternal alcohol consumption, maternal illicit drug use (*cannabis sativa*), maternal age, paternal age, breast feeding, birth order, chemical contamination in drinking water, both viral and bacterial infections, and parental cigarette smoking. [3]–[5] Alongside occupational exposure to benzene, [6] active tobacco smoking is an established risk for adult myeloid leukemia. [7] According to the International Agency for Research on Cancer (IARC), the available body of evidence suggests a consistent association of childhood leukemia with preconception and with combined paternal and maternal smoking. [7] Conversely, studies on maternal tobacco smoking often showed modest increases in risk, or null or inverse associations. [7] Only one study was included on second hand smoke and leukemia (namely chronic lymphocytic leukemia) reporting a positive association. [7] Most of the evidence on the relationship between cigarette smoking and childhood leukemia regards ALL, [8], while there is scant evidence for AnLL. [7], [9] As shown in supplemental Table S1, several studies highlighted that paternal smoking around the time of conception is a risk factor for childhood ALL. A meta-analysis of heavy paternal smoking (20+ cigarettes/day) highlighted a substantial increase in the risk of childhood leukemia (OR 1.44, 95%CI 1.24–1.68) [8].

Our aim was to investigate parental cigarette consumption and second-hand smoke exposure as risk factors for childhood AnLL, using data collected in a large case-control study primarily designed to evaluate the role of physical agents (including electromagnetic fields), parental occupation and environmental exposure in childhood hematopoietic malignancies. [10]–[11]

## Methods

### Study population

SETIL (*Studio sulla Eziologia dei Tumori Infantili Linfoemopoietici*, study on the etiology of childhood lympho-hematopoietic malignancies) is a population-based case-control study conducted in Italy between 1998 and 2003. Details of the study have been given elsewhere. [10]–[11] Thanks to the support of the Italian Association of Pediatric Hematology and Oncology almost all incident cases of childhood acute leukemia (aged between 0 and 10) in 14 Italian Regions were collected; [12] second primary neoplasms were excluded. Cases were individually matched for date of birth, sex and residence area with 2 population controls randomly drawn from Local Health Authority registries. Parents of eligible cases were contacted through the pediatric oncologist, parents of controls through their general practitioner; eligible subjects were asked to participate in a direct interview (non responders were 8% among cases and 29% among controls). During the study period 82 cases of AnLL, 601 cases of ALL and 1,044 controls (128 matched to AnLL cases and 916 matched to ALL cases) were enrolled.

Information was collected from parents of cases and controls in a direct interview using a standardized questionnaire that was constructed to collect data on many putative causes of childhood leukemia, including personal characteristics and exposure to physical, chemical and biological agents. For practical reasons, interviewers were not blinded to the case or control status of the child.

In the present analysis of AnLL, we broke the individual matching, and included the 82 cases of AnLL and all 1,044 sampled controls (irrespectively of individual matching with AnLL or ALL cases). Matching was retained in additional sensitivity analyses.

The SETIL study was conceived to investigate the etiology of hematopoietic malignancies. Findings on the association between tobacco smoke and risk of childhood acute lymphoblastic leukemia have been recently reported. [13] Queries about collaborations and access to the data can be addressed to the principal investigator of the SETIL Study (Prof. Corrado Magnani; email: magnani@med.unipmn.it). The SETIL study participated in the Childhood Leukemia International Consortium (CLIC, https://clic.berkeley.edu/about). [14]

The SETIL study was authorized by the ethics committee for the Piedmont Region (authorization n.2886, on 15/2/1999; letter n. 1852/28.3 on 17/2/1999) and later by the corresponding board of each participating research unit. Written informed consent was obtained from all participating subjects. The ethics committee approved the consent procedure.

### Exposure variables and covariates

An English language translation of the smoking sections of the SETIL questionnaire is presented in appendix S1. Available information on paternal smoking status in the period of conception enabled us to classify fathers in four categories: never a smoker; former smoker; smoker, 1 to 10 cigarettes per day; and smoker, 11 or more cigarettes per day. Based on preliminary analyses, never smokers and former smokers were merged, creating the category of non-smokers with reference to the period of conception. Information on the smoking status of fathers (smoker or non-smoker) was also available for the pregnancy and the period between birth and diagnosis. As expected, an excellent agreement (Cohen's kappa  = 0.96) was found between paternal smoking status in the conception period and smoking status after the child's birth.

For maternal smoking, information was available separately for each trimester of pregnancy. Since the consumption of cigarettes tended to be stable across the pregnancy (Cohen's kappa between first and third trimester  = 0.92), smoking status was classified according to the first trimester of pregnancy. After a preliminary analysis and considering the small numbers of active smokers — only three mothers of cases declared they had smoked more than 10 cigarettes/day — a dichotomous variable was created: non-smoker (never a smoker or former smoker); smoker. Mothers were asked to declare how many hours per day they had been exposed to Environmental Tobacco Smoking (ETS) during pregnancy. A three-level variable was created using the collected information: never exposed to passive smoking, and two levels of exposure based on the median of exposure to passive smoking among controls' mothers.

Exposure of children to ETS, measured in cigarettes per day, was collected for every year of life; Hence, we created a cumulative exposure index equal to the number of cigarettes to which the children had been exposed (ETS). Again, a three-level variable was created: never exposed to ETS, and two levels of exposure based on the median of exposure to passive smoking among controls.

Possible confounders were selected *a-priori* and included: sex, age group (less than two years; between two and four years; between four and six years; more than six years), residence area (part of Italy: North, except Lombardy; Lombardy; center; South and islands), birth order; birth weight; duration of breastfeeding; maternal and paternal age at child's birth; maternal and paternal education level; and parental occupational exposure to benzene. Exposure to benzene was assessed by industrial hygienists on the basis of information gathered with a job specific questionnaire. Detailed methods for the evaluation of exposure to benzene were presented in Miligi et al. [10]

### Statistical Analysis

Unmatched analyses were performed in order to avoid the loss of cases (9 cases were in matching strata without controls). To increase statistical power, considering that the sampling procedure and collection of information were the same for controls matched to AnLL and to ALL cases, we included all the 1,044 enrolled controls in the analysis and not only the 128 individually matched with AnLL cases. Unmatched analyses models always included age, gender and residence area. Matching was retained in additional sensitivity analyses.

In contingency tables, statistical independence of variables was tested using χ2 test or Fisher exact test, according to Cochran rule. [15] We examined associations between AnLL and each of the aforementioned sources of exposure to tobacco smoke. Odds Ratios (OR) and relative 95% Confidence Intervals (95% CI) were obtained with unconditional logistic regression models. Linear trends for ordinal exposure variables were evaluated using the Wald test, treating the variable as a continuous variable (introduced in the model with 1 degree of freedom). To test for possible interactions on a multiplicative scale, product terms for the interaction between the exposure variable and the proposed effect modifier were created and likelihood ratio tests were used to compare models with and without the interaction terms.

The limited number of cases (n = 82) did not allow the direct inclusion of all covariates in multivariate logistic regression models. To deal with the small number of events per parameter, we performed two separate sets of analyses. Firstly, we adjusted for putative confounders (parameterized as presented in Table 1) via inverse probability weighting (IPW). [16] Then the conditional probability of being exposed given the individual covariates were estimated by fitting probit (for dichotomous exposure) or multinomial probit (for categorical exposure) regression models and we calculated robust standard error for the inference. [16]–[18]. A second set of regression models including covariates selected based on the change-in-estimates methods were fitted, using a threshold for inclusion of a 10% change in the odds ratios of interest [19]. All analyses were performed using Stata 12.1 SE (Stata corporation, Texas, TX) and all tests were 2 sided. A p-value of 0.05 or less was considered statistically significant.

**Table 1:** Characteristics of Acute Non-Lymphocytic Leukemia Cases and Controls in the SETIL Case-Control Study, Italy, 1998-2003.

	AnLL cases		Controls matched to AnLL cases		All sampled controls		*P* value^a^
	No.	%	No.	%	No.	%	
Gender^d^							
Female	39	47.6	62	48.4	482	46.2	
Male	43	52.4	66	51.6	562	52.8	Na
Age at study reference date (years)^d^							
≤1	21	25.6	35	27.3	156	14.9	
2–3	13	15.9	18	14.1	351	33.6	
4–5	13	15.9	15	11.7	233	22.3	
≥6	35	42.7	60	46.9	304	29.1	Na
Residence area (part of Italy)^d^							
North (except Lombardy)	22	26.8	33	25.8	250	24.0	
Lombardy	16	19.5	32	25.0	260	24.9	
Center	17	20.7	23	18.0	257	24.6	
South and islands	27	32.9	40	31.2	277	26.5	Na
Birth order							
First born	39	47.6	68	53.1	551	52.8	
Second born	31	37.8	39	30.5	379	36.3	
Third born and others	12	14.6	21	16.4	113	10.8	0.49^b^
Birth weight (g)							
<3,000	19	23.2	31	24.2	239	22.9	
3,000–3,299	18	22.0	28	21.9	246	23.6	
3,300–3,599	23	28.0	29	22.7	254	24.4	
≥3,600	22	26.8	40	31.2	304	29.2	0.89^b^
Duration of breastfeeding (months)							
0	12	14.6	26	20.5	232	22.3	
1–3	32	39.0	32	25.2	267	25.7	
4–6	19	23.2	37	29.1	233	22.4	
>6	19	23.2	32	25.2	308	29.6	0.04^b^
Maternal age at child's birth (years)							
≤24	14	17.1	23	18.1	140	13.4	
25–29	25	30.5	41	32.3	382	36.7	
30–34	30	36.6	44	34.6	359	34.5	
≥35	13	15.8	19	15.0	160	15.4	0.65^b^
Birth year of the mother							
<1960	15	18.3	22	17.3	145	13.9	
1960–1964	27	32.9	32	25.2	328	31.5	
1965–1969	22	26.8	53	31.7	373	35.8	
≥1970	18	22.0	20	15.8	195	18.7	0.36^b^
Maternal educational level							
Less than high school	46	56.1	45	35.2	400	38.4	
High school	26	31.7	64	50.0	503	48.3	
University	10	12.2	19	14.8	139	13.3	<0.01^b^
Paternal age at child's birth (years)							
≤24	3	3.7	6	4.7	33	3.3	
25–29	18	22.2	29	22.8	241	23.8	
30–34	3	40.7	45	35.4	385	38.0	
≥35	27	33.3	47	37.0	353	34.9	0.96^b^
Paternal educational level							
Less than high school	47	58.0	54	42.2	463	44.6	
High school	24	29.6	59	46.1	424	40.9	
University	10	12.4	15	11.7	151	14.6	0.06^b^
Parental occupational exposure to benzene							
Absent	80	97.6	126	98.4	1,009	96.7	
Present	2	2.4	2	1.6	35	3.3	0.65^c^

Abbreviations: AnLL, acute non-lymphocytic leukemia; NA, not appropriate.

a Comparison between cases and all controls sampled in the SETIL study (AnLL controls + ALL controls).

b *P* values from χ2 test.

c *P* values from Fisher exact test.

d Matching variables.

## Results

Characteristics of study participants by case-control status are reported in Table 1. The entire sample of controls, mainly consisting of subjects matched to ALL cases, has a different age distribution compared to AnLL cases and their matched controls. The duration of breastfeeding was comparable in AnLL cases and their matched controls; conversely, long breastfeeding periods were more frequent in the control sample. Parents of cases usually had a lower educational level than controls' parents. All other considered characteristics seemed to have comparable distribution among cases and controls.

The ORs for the association between exposures to tobacco smoke and risk of AnLL are presented in Table 2. Estimates for both the matched and unmatched analyses are reported. In the unmatched analysis, ORs were estimated with reference to the subpopulation with complete data on putative confounders. Depending on the studied exposure, this restriction determined the exclusion of 33–39 controls and, only for paternal smoking in the conception period, of one case. Estimates based on the entire sample were consistent with those presented in Table 2.

**Table 2:** Association Between Acute non-Lymphocytic Leukemia and Sources of Exposure to Tobacco Smoke. The SETIL Study, Italy, 1998–2003.

					Unmatched analysis									
	Matched analysis^a^				Crude estimates				Models adjusted by sex, age and residence area		Models selected through change-in-estimates strategy		Models weighted by the inverse probability of exposure^f^	
Exposure	Ca	Co	OR	95%CI	Ca	Co	OR	95%CI	OR	95%CI	OR	95%CI	OR	95%CI
**Paternal smoking in the conception period**														
Non smoker	38	80	1.00	Ref.	38	612	1.00	Ref.	1.00	Ref.	1.00^b^	Ref.^b^	1.00	Ref.
Smoker, 1–10 cigs/day	12	15	1.95	0.76–5.04	12	123	1.57	0.80–3.09	1.74	0.87–3.48	1.59^b^	0.80–3.18^b^	1.34	0.65–2.76
Smoker, ≥11 cigs/day	30	33	1.76	0.91–3.41	30	264	1.83	1.11–3.02	1.90	1.14–3.17	1.79^b^	1.07–3.00^b^	1.79	1.01–3.15
*P*_trend_				0.09				0.02		0.01		0.02		0.05
**Maternal smoking in the 1^st^ trimester of pregnancy**														
Non smoker	71	115	1.00	Ref.	70	893	1.00	Ref.	1.00	Ref.	1.00^c^	Ref.^c^	1.00	Ref.
Smoker	11	14	1.22	0.47–3.12	11	111	1.26	0.65–2.46	1.35	0.68–2.66	1.30^c^	0.66–2.56^c^	0.83	0.38–1.81
**Maternal exposure to ETS during the pregnancy**														
Not exposed	49	84	1.00	Ref.	48	692	1.00	Ref.	1.00	Ref.	1.00^d^	Ref.^d^	1.00	Ref.
≤3 hours/day	15	22	0.99	0.43–2.29	15	188	1.15	0.63–2.10	1.03	0.55–1.92	1.04^d^	0.56–1.92^d^	0.89	0.46–1.72
>3 hours/day	17	19	1.69	0.78–3.64	17	115	2.13	1.18–3.83	1.94	1.06–3.54	2.12^d^	1.16–3.86^d^	1.85	0.97–3.52
*P*_trend_				0.23				0.02		0.06		0.03		0.07
**Cumulative exposure of child to ETS**														
Not exposed	52	89	1.00	Ref.	52	718	1.00	Ref.	1.00	Ref.	1.00^e^	Ref.^e^	1.00	Ref.
<4000 cigs	15	20	1.33	0.57–3.07	15	151	1.37	0.75–2.50	1.27	0.69–2.36	1.18^e^	0.64–2.18^e^	1.25	0.63–2.48
≥4000 cigs	15	20	1.59	0.65–3.87	14	130	1.49	0.80–2.76	1.51	0.78–2.92	1.33^e^	0.69–2.57^e^	1.15	0.45–2.95
*P*_trend_				0.29				0.15		0.18		0.39		0.77

Abbreviations: 95%CI, 95% confidence interval; cigs, cigarettes; ETS, environmental tobacco smoke; OR, odds ratio; Ref, reference category.

a Logistic regression models conditioned on matching variables (date of birth, sex, residence area of the child).

b Logistic regression model adjusted by age class and maternal educational level.

c Logistic regression model adjusted by age class and maternal educational level.

d Logistic regression model adjusted by duration of breastfeeding and paternal educational level.

e Logistic regression model adjusted by age class, maternal and paternal educational level.

f Logistic regression model adjusted sex, age class, residence area, birth order, birth weight, duration of breastfeeding, maternal and paternal age at child birth, maternal and paternal educational level, birth year of the mother, and parental occupational exposure to benzene (inverse probability weighting).

**Figure 1:**
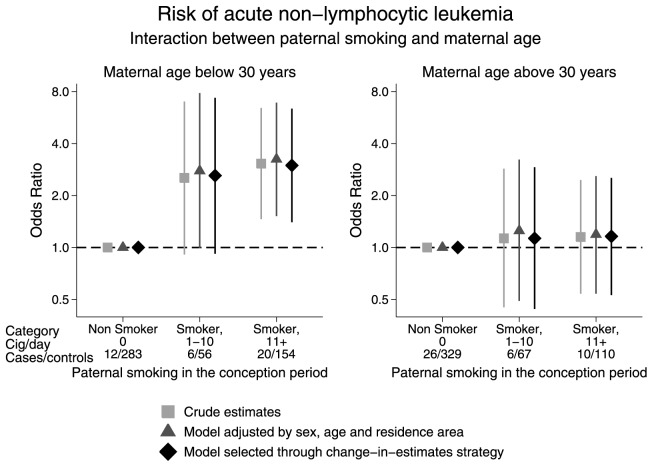
Association Between Paternal Smoking Status During the Period of Conception and Risk of Childhood Acute Non-Lymphocytic Leukemia, According to Maternal Age at Delivery. The SETIL Study, Italy, 1998–2003.

As shown in table 2, in matched analysis, paternal smoking in the conception period presented signs of association with the risk of AnLL (OR of smokers, 1–10 cigarettes/day  = 1.95, 95%CI 0.76–5.04; OR of smokers, 11 or more cigarettes/day  = 1.76, 95%CI 0.91, 3.41; *P* for trend 0.09). Unmatched analysis of paternal smoking produced similar estimates (adjusted OR of smokers, 1–10 cigarettes/day  = 1.34, 95%CI 0.65–2.76; OR of smokers, 11 or more cigarettes/day  = 1.79, 95%CI 1.01, 3.15; *P* for trend 0.05). Although supported by very weak statistical evidence (*P* = 0.18), the study of the interaction between paternal smoking and maternal age at child's birth showed interesting estimates (Figure 1). Apparently, paternal smoking affected the risk of childhood AnLL only among children born from mothers aged below 30 years, a cut-off selected *a priori* based on median maternal age. In the multivariable model selected with the change-in-estimate method and including age at diagnosis and maternal educational level, the adjusted OR for moderate smokers (1–10 cigarettes/day) was 2.61 (95%CI0.92–7.36), while the OR for heavy-smoker fathers (11 or more cigarettes/day) was 2.99 (95%CI1.40–6.37). Estimates for children born from mothers aged above 30 years were close to the unit (adjusted OR of smokers, 1–10 cigarettes/day  = 1.13, 95%CI0.44–2.92, OR of smokers, 11 or more cigarettes/day  = 1.16, 95%CI0.53–2.53). Of note, almost no evidence was found of an interaction between paternal age and paternal smoking during the conception period (at multivariate analysis p interaction  = 0.40, data not shown).

Maternal smoking during the first trimester of pregnancy did not show clear signs of association with the risk of childhood AnLL (Table 2). However, in unmatched analysis, marginal evidence of an association of AnLL with high levels of maternal exposure to ETS during the pregnancy (adjusted OR of mothers exposed more than 3 hours/day  = 1.85, 95%CI 0.97–3.52; *P* for trend  = 0.07) were observed. However, the exclusion of active-smoker mothers (n = 117) from the analysis determined a decrease of the estimates (adjusted OR of mothers exposed more than 3 hours/day  = 1.42, 95%CI 0.69–2.95). The further adjustment by paternal smoking (an exposure that is likely to be associated with maternal exposure to ETS) caused a modest increase of the estimates (adjusted OR of mothers exposed more than 3 hours/day  = 1.61, 95%CI 0.73–3.53).

As shown in Table 2, no evidence supported an association between the exposure of the child to ETS and the risk of AnLL (for children exposed to 4,000 or more cigarettes, OR adjusted through IPW  = 1.15, 95%CI 0.45–2.95; *P* for trend  = 0.77).

## Discussion

In this analysis of data from a population-based case-control study moderate evidence supporting the hypothesis that children of fathers who smoked in the period of conception have an increased risk of AnLL was found. Interestingly, an apparent effect modification by maternal age was also identified. Indeed, only children of mothers aged below 30 years at the delivery presented an increased risk. We also found weak signs of an association between maternal exposure to second-hand smoke and risk of childhood AnLL. No sign of association was found for maternal smoking during pregnancy. Finally, we did not find any evidence supporting an association between child exposure to second-hand smoke and risk of AnLL.

### Plausibility of the results and evidence from previous studies

An association between paternal smoking before the pregnancy and risk of childhood leukemia has already been reported. [7]–[9], [20] However, most of the positive findings regarded ALL, while only limited evidence supports the association between AnLL and paternal smoking. [7] It should be considered that studies on AnLL and paternal smoking are all case-control studies and they are often underpowered, due to the rarity of the disease. Since tobacco smoke is an established leukemogenic in adults, [7] the biological plausibility of an association with childhood AnLL is high. Furthermore, the possible effect of exposure to tobacco smoke of the gametes or the embryo/fetus in utero on the risk of childhood AnLL is in line with the “two hits” model proposed by Greaves. [21] Moderate/weak evidence of a possible interaction between paternal smoking and maternal age at delivery was observed. Possible explanations of the observed interaction could be chance or a strong pattern of confounders differentially acting in the two maternal age strata. However, further investigations should be carried out before excluding causality, since during pre-implantation embryogenesis complex interactions exist between paternal and maternal factors and the biochemical environment. [22]

Our analysis did not produce evidence supporting an association between maternal tobacco smoking and risk of childhood AnLL. Results were broadly in line with those of previous studies. [7] However, one should consider our sample only included 19 women (3 cases and 16 controls) who declared having smoked more than 10 cigarettes/day during the first trimester of pregnancy.

Results for maternal exposure to second-hand smoke suggest a possible association with AnLL: to the best of our knowledge, this finding is the first supporting this association [23], [24] which makes us cautious in interpreting this apparent association as causal since we consider the self-assessment of second-hand smoke to be a measure prone to misclassification and recall bias. In fact, the presence of a raised risk only for maternal exposure to ETS and not for maternal active smoking is difficult to explain from a biological point of view. Furthermore, evidence suggesting a strong recall bias for maternal exposure to ETS emerged from a former study of ALL performed data from the SETIL study [13].

In most past studies on exposure of children to second-hand smoke and risk of AnLL authors used parental smoking status after pregnancy as a proxy of exposure, and most findings were negative. [25] In the SETIL study, a quantification of child exposure was attempted with direct questions in the questionnaire, but we failed to find any sign of an association between second-hand smoke and AnLL risk.

### Strengths and limitations

One strength of this study is the population based design: the identification of incident cases in participating Regions proved to be very accurate [12] and information on exposures was collected by trained interviewers.

Conversely, several limitations should be considered: the response rate of controls was 0.71 and we cannot exclude a selection bias. Recall bias is always a concern when investigating self-reported exposures. Nevertheless, a Swedish study highlighted that retrospective recall of pregnancy smoking is fairly stable over time. [26] Also, interviewed subjects and interviewers were unaware of the hypothesis investigated in the present report since studying the association between smoking and childhood ALL was not one of the main purposes of the SETIL study; furthermore, the sections aimed at collecting information on smoking were only a small part of the entire questionnaire. On the balance, we do not believe that recall bias is a serious limitation for the study of parental active smoking; on the contrary, recall bias could affect the study of ETS. As the SETIL study was not primarily designed to study the effect of tobacco smoking, misclassification of exposure could be an issue, particularly for ETS exposure.

In the present analysis we were unable to consider the effect of residential and domestic exposure to benzene, possible confounders of the relationship between exposure to cigarette smoke and risk of childhood AnLL.

We decided to break the matching in order to avoid loss of cases and expand the control group. Therefore, we should consider a possible bias due to the use of unconditional logistic regression in analysis that involved both matched and unmatched controls, with respect to AnLL cases. Of note, estimates from conditional logistic regression models (matched analysis) were consistent with the results from unmatched analysis.

The use of a propensity score or inverse probability weighting in case-control studies has been reported to be more problematic than in cohort studies, since estimates might be affected by an artefactual effect modification and residual confounding [27]. To assess whether this sort of bias might influence our estimates a supplemental set of analyses where covariates were selected based on the change-in-estimates method was performed. It is noteworthy to observe that figures from the two sets of analyses were consistent.

## Conclusions

Our study supports the hypothesis that paternal smoking is associated with the risk of childhood AnLL; we also found signs of a possible effect modification due to maternal age at delivery that should be considered in future investigations. We found weak evidence of a possible effect of maternal exposure to second-hand smoke on the risk of childhood AnLL. This finding has to be consider with a degree of caution since recall bias is likely. No evidence at all emerged in our analysis for maternal smoking and exposure of the child to second-hand smoke; these results are broadly in line with knowledge from previous researches, but we should underline that the power of our study to detect an association for these exposures was low.

## Supporting Information

Table S1 Studies on Paternal Tobacco Smoking and Risk of Childhood Acute non-Lymphocytic Leukemia.
(DOCX)

Appendix S1 Smoking questionnaire.
(DOCX)
