# Supplementary material for: Tobacco Smoke and Risk of Childhood Acute Non-Lymphocytic Leukemia: Findings from the SETIL Study
Source: PLoS One. 2014 Nov 17;9(11):e111028. doi: 10.1371/journal.pone.0111028 (PMC4234298; doi:10.1371/journal.pone.0111028)
Supplement: Table S1 — Studies on Paternal Tobacco Smoking and Risk of Childhood Acute non-Lymphocytic Leukemia. (DOCX) [file pone.0111028.s001.docx]

**Table S1.** Studies on Paternal Tobacco Smoking and Risk of Childhood Acute non-Lymphocytic Leukemia.

| **Study** | **Cases/controls** | **Confounders/matching variables** | **Main results for paternal smoking** |
| --- | --- | --- | --- |
| Magnani et al, 1990 | 22/228 | Analysis adjusted for socioeconomic status | Paternal smoking up to the child’s birth (no/yes): OR 0.9 (95%CI 0.3–2.1) |
| Shu et al, 1996 | 79/?(≤158) | Matched analysis (birth year, telephone area code, and exchange number) adjusted for sex, paternal age, education, and maternal alcohol consumption during pregnancy | No. of cigarettes smoked during the month prior to pregnancy:  None: 1.00 (Ref.)  1-10: OR 0.42 (95%CI 0.09–1.95)  11-20: OR 0.73 (95%CI 0.27–1.94)  >20: OR 1.29 (95%CI 0.44–3.74) |
| Ji et al, 1997 | 52/52 | Matched analysis (age and sex) adjusted for birth weight, income, paternal age, education, and alcohol drinking. | Pack years before conception:  0 pack years: OR 1.0 (Ref.)  ≤2 pack years: OR 0.9 (95%CI 0.1-7.3)  2–5 pack years: OR 0.6 (95%CI 0.1-3.1)  ≥5 pack years: OR 2.3 (95%CI 0.4-14.8) |
| Sorahan et al, 1997a | 151/151 | None | Change of one level for daily cigarette consumption (nonsmokers, < 10 cpd, 10-19 cpd, 20-29 cpd, 30-39 cpd, and > 40 cpd): OR 1.02 (95%CI 0.89-1.16) |
| Sorahan et al, 1997a | 115/115 | Maternal smoking | Change of one level for smoking habit (nil, slight, moderate and heavy): OR 0.98 (95%CI 0.73-1.32) |
| Sorahan et al, 1997a | 190/190 | Maternal smoking | Change of one level for smoking habit (nil, slight, moderate and heavy): OR 1.27 (95%CI 1.10-1.47) |
| Brondum et al, 1999 | 450/523 | Matched analysis (age, race, telephone area code and exchange) and adjusted for annual income, father’s race and education | One month before pregnancy (no/yes): OR 0.87 (95%CI 0.64–1.18) |
| Pang et al, 2003 | 230/6987 | Analysis adjusted for sex, age, UKCCS region, parental age, and deprivation | Preconception (no/yes): OR 1.07 (95%CI 0.80 1.43) |
| Chang et al, 2006 | 39/44 | Matched analysis (age and sex) adjusted for household income | Ever/never: OR 2.64 (95%CI 0.98–7.12)  Preconception (no/yes): OR 3.84 (95%CI 1.04–14.17) |
| Menegaux et al, 2007 | 62/567 | Analysis adjusted for age, gender, region, socio-professional category, and birth order | Before pregnancy:  None: OR 1.0 (Ref.)  ≤10 cpd: OR 0.9 (95%CI 0.5–1.7)  >10 cpd: OR 0.2 (95%CI 0.02–1.7) |
| MacArthur et al, 2008 | 39/39 | Matched analysis (age, gender and region) adjusted for maternal age at birth, maternal education, household income, ethnicity, and number of residences since birth | Cigarettes/day before pregnancy:  None: OR 1.00 (Ref.)  <10: OR 2.98 (95%CI 0.70–12.75)  11-19: OR 0.93 (95%CI 0.25–3.45)  ≥20: OR 0.90 (95%CI 0.34–2.38) |
| Rudant et al, 2008 | 102/1681 | Analysis adjusted for age, gender, parental professional category and maternal age at the time of birth | Cigarettes/day from the year prior to the child’s birth to the interview:  None: OR 1.00 (Ref.)  <10: OR 1.4 (95%CI 0.7–2.9)  11-19: OR 1.3 (95%CI 0.7–2.4)  ≥20: OR 1.7 (95%CI 1.0–2.9) |
| Metayer et al, 2013 | 135/164 | Analysis adjusted for child's age at diagnosis/reference date, sex, and Hispanic status, maternal race, and household annual income | Paternal prenatal smoking (no/yes): OR 1.36 (95%CI 0.82–2.24) |

Abbreviations: 95%CI, 95% confidence intervals; cpd, cigarettes per day; OR, odds ratio

**References**

- Brondum J, Shu XO, Steinbuch M, Severson RK, Potter JD, Robison LL (1999) Parental cigarette smoking and the risk of acute leukemia in children. Cancer 85:1380 - 1388.
- Chang JS, Selvin S, Metayer C, Crouse V, Golembesky A, Buffler PA (2006) Parental smoking and the risk of childhood leukemia. Am J Epidemiol 163:1091 - 1100.
- Ji BT, Shu XO, Linet MS, Zheng W, Wacholder S, Gao YT, Ying DM, Jin F (1997) Paternal cigarette smoking and the risk of childhood cancer among offspring of nonsmoking mothers. J Natl Cancer Inst 89:238 - 244.
- MacArthur AC, McBride ML, Spinelli JJ, Tamaro S, Gallagher RP, Theriault G (2008) Risk of childhood leukemia associated with parental smoking and alcohol consumption prior to conception and during pregnancy: the cross-Canada childhood leukemia study. Cancer Causes Control 19:283 - 295.
- Magnani C, Pastore G, Luzzatto L, Terracini B (1990) Parental occupation and other environmental factors in the etiology of leukemias and non-Hodgkin's lymphomas in childhood: a case-control study. Tumori 31;76:413-419.
- Menegaux F, Ripert M, Hémon D, Clavel J (2007) Maternal alcohol and coffee drinking, parental smoking and childhood leukaemia: a French population-based case-control study. Paediatr Perinat Epidemiol 21:293 - 299.
- Metayer C, Zhang L, Wiemels JL, Bartley K, Schiffman J, Ma X, Aldrich MC, Chang JS, Selvin S, Fu CH, Ducore J, Smith MT, Buffler PA (2013) Tobacco smoke exposure and the risk of childhood acute lymphoblastic and myeloid leukemias by cytogenetic subtype. Cancer Epidemiol Biomarkers Prev 22:1600 - 1611.
- Pang D, McNally R, Birch JM (2003) Parental smoking and childhood cancer: results from the United Kingdom Childhood Cancer Study. Br J Cancer 88:373 - 381.
- Rudant J, Menegaux F, Leverger G, Baruchel A, Lambilliotte A, Bertrand Y, Patte C, Pacquement H, Vérité C, Robert A, Michel G, Margueritte G, Gandemer V, Hémon D, Clavel J (2008) Childhood hematopoietic malignancies and parental use of tobacco and alcohol: the ESCALE study (SFCE). Cancer Causes Control 19:1277 - 1290.
- Sorahan T, Lancashire R, Prior P, Peck I, Stewart A (1995) Childhood cancer and parental use of alcohol and tobacco. Ann Epidemiol 5:354-9.
- Sorahan T, Lancashire RJ, Hultén MA, Peck I, Stewart AM (1997) Childhood cancer and parental use of tobacco: deaths from 1953 to 1955. Br J Cancer 75:134 - 138.
- Sorahan T, Prior P, Lancashire RJ, Faux SP, Hultén MA, Peck IM, Stewart AM (1997) Childhood cancer and parental use of tobacco: deaths from 1971 to 1976. Br J Cancer 76:1525 - 1531.
- Shu XO, Ross JA, Pendergrass TW, Reaman GH, Lampkin B, Robison LL (1996) Parental alcohol consumption, cigarette smoking, and risk of infant leukemia: a Childrens Cancer Group study. J Natl Cancer Inst 88:24 - 31.
