# Supplementary material for: Tobacco Smoke and Risk of Childhood Acute Non-Lymphocytic Leukemia: Findings from the SETIL Study
Source: PLoS One. 2014 Nov 17;9(11):e111028. doi: 10.1371/journal.pone.0111028 (PMC4234298; doi:10.1371/journal.pone.0111028)
Supplement: Appendix S1 — Smoking questionnaire. (DOCX) [file pone.0111028.s002.docx]

**Appendix S1.** Smoking questionnaire.

| **MATERNAL SMOKING** | | | | | | | | |  | | | |  | | | |  | | | | | |
| --- | --- | --- | --- | --- | --- | --- | --- | --- | --- | --- | --- | --- | --- | --- | --- | --- | --- | --- | --- | --- | --- | --- |
| **Active smoking** | | | | | | | | |  | | | |  | | | |  | | | | | |
| 1) Have you ever smoked (at least 1 cigarette/day for 6 months)? | | | | | | | | | 🞎 Yes | | | | 🞎 No | | | | 🞎 Don’t know | | | | | |
| 2) If yes, in which year did you start smoking? _____________ | | | | | | | | |  | | | |  | | | |  | | | | | |
| 3) Do you currently smoke? | | | | | | | | | 🞎 Yes | | | | 🞎 No | | | | 🞎 Don’t know | | | | | |
| 4) If you quit smoking, when was that (year)? _____________ | | | | | | | | |  | | | |  | | | |  | | | | | |
| 5) Did you smoke during your pregnancy? | | | | | | | | | 🞎 Yes | | | | 🞎 No | | | | 🞎 Don’t know | | | | | |
| If yes, please specify: | | | | | | | | |  | | | |  | | | |  | | | | | |
| 5.1) 1^st^ trimester | | | | | | | | | 🞎 Yes | | | | 🞎 No | | | | Cigarettes/day­­_____ | | | | | |
| 5.2) 2^nd^ trimester | | | | | | | | | 🞎 Yes | | | | 🞎 No | | | | Cigarettes/day_____ | | | | | |
| 5.3) 3^rd^ trimester | | | | | | | | | 🞎 Yes | | | | 🞎 No | | | | Cigarettes/day_____ | | | | | |
| **Secondhand smoke** | | | | | |  | | | |  | | | | | |  | | | | | | |
| 1) During the pregnancy, have you been exposure to secondhand smoke (at least one hour per day)? | | | | | | 🞎 Yes | | | | 🞎 No | | | | | | 🞎 Don’t know | | | | | | |
| 2) If yes, where? | | | | | | 🞎 At home | | | | | | 🞎 At work | | | | | | | 🞎 Other places | | | |
| 3) On average, how many hours per day have you been exposed? | | | | | |  | | | |  | | | | | |  | | | | | | |
| 3.1) During the workweek (Monday-Friday) | | | | | | Hours/day­­_____ | | | | | | | | | | | | | | | | |
| 3.2) During the weekend (Saturday-Sunday) | | | | | | Hours/day_____ | | | | | | | | | | | | | | | | |
|  | | | | | |  | | | | | | | | | | | | | | | | |
| **PATERNAL SMOKING** | | | | | | | | |  | | | |  | | | |  | | | | | |
| **Active smoking** | | | | | | | | |  | | | |  | | | |  | | | | | |
| 1) Have you ever smoked (at least 1 cigarette/day for 6 months)? | | | | | | | | | 🞎 Yes | | | | 🞎 No | | | | 🞎 Don’t know | | | | | |
| 2) If yes, in which year did you start smoking? _____________ | | | | | | | | |  | | | |  | | | |  | | | | | |
| 3) Do you currently smoke? | | | | | | | | | 🞎 Yes | | | | 🞎 No | | | | 🞎 Don’t know | | | | | |
| 4) If you quit smoking, when was that (year)? _____________ | | | | | | | | |  | | | |  | | | |  | | | | | |
| 5) Did you smoke during the conception period or the pregnancy? | | | | | | | | | 🞎 Yes | | | | 🞎 No | | | | 🞎 Don’t know | | | | | |
| If yes, please specify: | | | | | | | | |  | | | |  | | | |  | | | | | |
| 5.1) During the conception period | | | | | | | | | 🞎 Yes | | | | 🞎 No | | | | 🞎 Don’t know | | | | | |
| If yes, cigarettes/day­­_____ | | | | | | | | |  | | | |  | | | |  | | | | | |
| 5.2) During the pregnancy | | | | | | | | | 🞎 Yes | | | | 🞎 No | | | | 🞎 Don’t know | | | | | |
| If yes, cigarettes/day­­­_____ | | | | | | | | |  | | | |  | | | |  | | | | | |
|  | | | | | | | | |  | | | |  | | | |  | | | | | |
| **EXPOSURE OF THE CHILD TO SECONDHAND SMOKE** | | | | | | | | | | | | | | | | | | | | | | |
| Has someone ever smoke in the same room as the child? | | | | | | | 🞎 Yes | | | | | | | 🞎 No | | | | | | 🞎 Don’t know | | |
| If yes, please specify: | | | | | | | | | | | | | | | | | | | | | | |
|  | Age (completed years) | | | | | | | | | | | | | | | | | | | | | |
|  | 0 | 1 | 2 | 3 | 4 | | | 5 | | | 6 | | | | 7 | | | 8 | | | 9 | 10 |
| Never |  |  |  |  |  | | |  | | |  | | | |  | | |  | | |  |  |
| Occasionally (1 cigarette/day) |  |  |  |  |  | | |  | | |  | | | |  | | |  | | |  |  |
| 1-10 cigarettes/day |  |  |  |  |  | | |  | | |  | | | |  | | |  | | |  |  |
| 11+ cigarettes/day |  |  |  |  |  | | |  | | |  | | | |  | | |  | | |  |  |
